# The association between methylphenidate treatment and the risk for fracture among young ADHD patients: A nationwide population-based study in Taiwan

**DOI:** 10.1371/journal.pone.0173762

**Published:** 2017-03-15

**Authors:** Vincent Chin-Hung Chen, Yao-Hsu Yang, Yin-To Liao, Ting-Yu Kuo, Hsin-Yi Liang, Kuo-You Huang, Yin-Cheng Huang, Yena Lee, Roger S. McIntyre, Tzu-Chin Lin

**Affiliations:** 1 Chang Gung Medical Foundation, Chiayi Chang Gung Memorial Hospital, Chiayi County, Taiwan; 2 Department of Medicine, School of Medicine, Chang Gung University, Tao-Yuan, Taiwan; 3 Department of Traditional Chinese Medicine, Chang Gung Memorial Hospital, Chia-Yi, Taiwan; 4 Center of Excellence for Chang Gung Research Datalink, Chang Gung Memorial Hospital, Chiayi, Taiwan; 5 Institute of Occupational Medicine and Industrial Hygiene, National Taiwan University College of Public Health, Taipei, Taiwan; 6 School of Traditional Chinese Medicine, College of Medicine, Chang Gung University, Taoyuan, Taiwan; 7 Department of Psychiatry, Chung Shan Medical University Hospital, Taichung, Taiwan; 8 Department of Psychiatry, School of Medicine, Chung Shan Medical University, Taichung, Taiwan; 9 Department of Child Psychiatry, Linkou Chang Gung Memorial Hospital, Taoyuan, Taiwan; 10 Department of Speech, Language Pathology and Audiology, Chung Shan Medical University, Taichung, Taiwan; 11 Department of Neurosurgery, Chang Gung Memorial Hospital, Chiayi; 12 Mood Disorders Psychopharmacology Unit, University Health Network, University of Toronto, Toronto, Canada; 13 Department of Psychiatry, University of Toronto, Toronto, Canada; University of Nottingham, UNITED KINGDOM

## Abstract

Attention-deficit hyperactivity disorder (ADHD) is associated with higher risk for fracture. Whether the medical treatment for ADHD would mitigate the risk remains unclear. In this study, we sought to investigate the effect of methylphenidate treatment on risk for fracture, as well the moderational role of treatment duration on the risk of fracture, in a large national sample. Cases less than 18 years old were identified from Taiwan’s National Health Insurance Research Database with a new primary diagnosis of ADHD (ICD-9:314) between 1996 and 2013. A total of 6201 cases with ADHD were included as the study cohort. The cases were divided into 3 groups according to the duration of methylphenidate treatment (0, 1–180, and more than 180 days). All groups were followed until the end of 2013 for first diagnoses of fracture (ICD-9 codes 800 to 829). Cox proportional hazards models were applied. Compared to the group without methylphenidate treatment, the risk for fracture was lower among the group treated for more than 180 days. The adjusted hazard ratio was 0.77 (95% Confidence interval: 0.63–0.94). The groups treated for 180 days or fewer had no significant difference in the risk for fracture. In conclusion, methylphenidate treatment was associated with lower risk for fracture among ADHD patients. The association was evident only in the cohort treated for more than 180 days.

## Introduction

Attention-deficit hyperactivity disorder (ADHD) is a neuropsychiatric condition characterized by hyperactivity, impulsivity, and cognitive dysfunction [1]. The typical age at onset of ADHD is during childhood and adolescence with an estimated 10–30% of affected individuals continuing to manifest symptoms during adulthood [2, 3]. The global lifetime prevalence of ADHD is estimated to be 5.29% [4]. Patients with ADHD often have significant impairment in academic functioning as well as in social and interpersonal domains [5–7]. In addition, accumulating evidence indicates that ADHD is associated with increased risk for accidental physical injuries, such as motor vehicle accidents and traumatic brain injuries [8–12]. The core symptoms of ADHD including inattention, distractibility, and impulsivity likely account for the increased risk for unintentional self-harm [13].

The impact of ADHD medication also received growing attention as well. The study by van den Ban et al revealed that the incidence rate for injuries during exposure to ADHD drugs was lower in the exposed period compared to the period prior to ADHD drug use [14]. Another self-controlled case series study demonstrated that the ADHD medication had a preventive effect on the risk of brain injuries (34% risk reduction) [15]. Furthermore, the study by Lange et al reported that ADHD-affected youngsters had higher risks for accidents than their unaffected counterparts and the medication intake was only a weak predictor for accidents [16]. These studies tried to explore the role of ADHD medication on the risk for different kinds of injuries. These results could not be compared directly owing to the different study designs and different outcomes (e.g. injuries, brain injuries, and accidents).

Within the broad category of unintentional self-harm or injuries among children and adolescent, fracture is a well-defined diagnosis. The injuries from fracture are common and further add to the illness-associated morbidity in ADHD [17–21]. Two recently published population-based studies in Taiwan provide empirical evidence that, in the general population, ADHD is highly associated with fracture in children and adolescents [22, 23]. Notwithstanding the association between ADHD and risk of unintentional injury, relatively few studies have evaluated whether exposure to psychostimulant treatment mitigates the risk. Moreover, it is also not known whether the duration of psychostimulant exposure moderates the possible injury-lowering effects of psychostimulants.

Despite unequivocal evidence documenting efficacy across core symptoms of ADHD [24], insufficient medical treatment duration is a common problem resulting in inadequate treatment response among ADHD cases [25–27]. The National survey in Taiwan by Gau et al also indicated that poor adherence was associated with more severe ADHD-related symptoms [28]. The high rate of treatment non-adherence to psychostimulants invites the need to clarify whether the duration of exposure to stimulants mitigates any salutary effects.

Herein, we primarily aimed to determine, by using a nationwide population-based dataset in Taiwan, the effect of psychostimulant prescription for ADHD on mitigating the risk of fracture, and whether the mitigating effects are moderated by the duration of exposure among cases less than 18 years old.

## Materials and methods

### Sample

This retrospective cohort study utilized data from the Taiwan National Health Insurance Research Database (NHIRD) under the aegis of the National Health Research Institute (NHRI) which included outpatient, ambulatory, hospital inpatient care, as well as dental services. The National Health Insurance (NHI) program provides compulsory universal health insurance, implemented in March 1995, covering all delivery of health care in 99.5% of the national population. In cooperation with the Bureau of NHI, the NHRI extracted a randomly sampled representative database of 1,000,000 people from the registry of all NHI enrollees using a systematic sampling method for research purposes, forming the Longitudinal Health Insurance Database (LHID). There are no statistically significant differences in age, sex, or health care costs between this sample and all enrollees [29].

ADHD cases were identified based on recorded International Classification of Disease, Ninth revision (ICD-9) codes of 314. All medical claims made under this diagnostic code between 1996 and 2013 were collected from NHIRD for further analysis. The definition of ADHD for this analysis required an inpatient diagnosis or two outpatient diagnoses during one year. Using the foregoing definition, 9826 ADHD cases between 1996 and 2013 were identified. Cases born before 1996 or after 2005, fracture before ADHD diagnosis, missing residential data, or ADHD diagnosis within one year prior to study period were excluded. Total 6201 cases of ADHD comprised the final study cohort.

In Taiwan, methylphenidate was the only stimulant approved for ADHD treatment during 1996 to 2013, including short- and sustained-release preparations The two preparations of methylphenidate had similar effects on ADHD symptoms, but the sustained-release preparation was more convenient with lesser rebound side effects [30, 31]. Atomoxetine, a non-stimulant, was also approved as a medication for ADHD in Taiwan since 2007. The approved ages for both methylphenidate and atomoxetine are 6 and older in Taiwan. Methylphenidate is recommended by the Taiwan National Health Insurance as a first-line treatment for ADHD. Atomoxetine is recommended for the treatment of ADHD in cases where methylphenidate treatment results in insufficient treatment outcomes (i.e. inefficacy, intolerability). It can be reasonably assumed that atomoxetine exposures have had prior exposure to methylphenidate treatment. As atomoxetine is not a psychostimulant and we were primarily interested in psychostimulant risk mitigation, we confined our analysis to those cases that were prescribed with methylphenidate only. Cases with both methylphenidate and Atomoxetine prescriptions had been excluded. Furthermore, ADHD cases in Taiwan might have received both immediate and sustained-release preparations of methylphenidate during different periods of their clinical course. Therefore, we sought to determine the influence of the duration of methylphenidate exposure, regardless of their pharmacological formulations, by evaluating groups according to three separate duration intervals.

The treatment intervals chosen were 0, 1–180, and more than 180 days respectively. It was based on several epidemiological studies revealing that most ADHD cases received medical treatment for less than 180 days [32–34]. The effects of treatment duration exceeding 180 days deserved exploration. The treatment duration was defined as the cumulative length of methylphenidate exposure (days) within the follow-up time until fracture, death, or end of study period. The length of exposure was cumulated whether the prescriptions were continuous or interrupted. All groups were followed for incidence of fracture as an outcome, which was defined on the basis of ICD-9 codes 800–829. Individuals with fracture were identified if he/she had two or more outpatient diagnoses in the same year or any inpatient diagnosis.

Covariates considered in this analysis were chosen a priori on the basis of hypothetical associations with the exposure and outcome of interest. These comprised several demographic factors such as sex, age and urbanized level of residence [35]. Comorbid conditions associated with the risk for fracture were also considered, including seizure (ICD-9 code 345) [36–38], asthma (ICD-9 code 493) [39–42], intellectual disability (ICD-9 codes 317–319) [43, 44], autism (ICD-9 code 299) [45, 46], benzodiazepine (BZD) or hypnotic use[47], and conduct disorder (ICD-9 code 312) [48]. The definitions of asthma, seizure, intellectual disability, autism and conduct disorder were based on two or more outpatient diagnoses in the same year or any inpatient diagnosis. The BZD/hypnotic use referred to any prescription of the medications (ATC codes N05B, N05C) during the follow-up period.

The NHIRD consists of de-identified secondary data released to the public for research purposes. This study had been reviewed and approved by the Institutional Review Board of Chung Shan Medical University Hospital.

### Statistical analysis

The distribution of demographic factors and the proportions of comorbidities between the three groups with different treatment duration of methylphenidate were compared. Cox proportional hazards models were used to compute the hazard ratios (HRs) accompanying 95% confidence intervals (CIs) after adjustment for sex, age, urbanized level of residence, seizure, asthma, intellectual disability, autism, BZD/hypnotic use, and conduct disorder. Two-tailed P = 0.05 was considered significant. Patients with a death date in the admission file and those withdrawn from the registry for beneficiaries were censored. All of these analyses were conducted using SAS statistical software (Version 9.4; SAS Institute, Cary, NC, USA).

## Result

### Characteristics of subjects

The study cohort comprised 6201 cases with ADHD. The characteristics of the three groups with different treatment duration of methylphenidate are described and compared in Table 1.

**Table 1:** Characteristics of groups with different treatment duration of methylphenidate.

Variables	Methylphenidate						
	0 day		1–180 days		>180 days		
	(N = 2,623)		(N = 1,742)		(N = 1,836)		
	count	(%)	count	(%)	count	(%)	P value
**Sex**							< .001
Female	664	(25.3%)	338	(19.4%)	301	(16.4%)	
Male	1,959	(74.7%)	1,404	(80.6%)	1,535	(83.6%)	
**Age**	6.90 ± 2.69		8.10 ± 2.65		7.53 ± 2.42		< .001
0–5	856	(32.6%)	242	(13.9%)	308	(16.8%)	
6–11	1,602	(61.1%)	1,293	(74.2%)	1,387	(75.5%)	
12–18	165	(6.3%)	207	(11.9%)	141	(7.7%)	
**Urbanized level of residence**							
1 (City)	983	(37.5%)	592	(34.0%)	665	(36.2%)	< .001
2	1,262	(48.1%)	829	(47.6%)	842	(45.9%)	
3	285	(10.9%)	210	(12.1%)	215	(11.7%)	
4 (Villages)	93	(3.5%)	111	(6.4%)	114	(6.2%)	
**Covariates**							
** Seizure**							
	82	(3.1%)	60	(3.4%)	76	(4.1%)	0.192
** Asthma**							
	834	(31.8%)	550	(31.6%)	610	(33.2%)	0.500
** Intellectual disability**							
	126	(4.8%)	98	(5.6%)	117	(6.4%)	0.075
** Autism**							
	115	(4.4%)	78	(4.5%)	73	(4.0%)	0.724
** Conduct disorder**							
	55	(2.1%)	57	(3.3%)	70	(3.8%)	0.002
** BZD/hypnotic use**							
	767	(29.2%)	527	(30.3%)	586	(31.9%)	0.160
**Fracture**							
	248	(9.5%)	196	(11.3%)	159	(8.7%)	0.027
**Follow-up duration (year)**	5.33 ± 3.20		4.90 ± 3.02		5.75 ± 2.79		< .001

The differences of demographic characters between three groups were sex, age, urbanized level of residence, and follow-up duration. The group with medication for more than 180 days had the highest male/female ratio (83.6%/16.4%). Most cases were between 6–11 years old and the mean age was lowest in the group without methylphenidate treatment (6.90± 2.69). Most cases were from the area of moderate to high level of urbanization and the group without methylphenidate treatment had the least cases from the least urbanized area (3.5%). The follow-up duration was longest in the group with methylphenidate treatment for more than 180 days (5.75 ± 2.79 years).

Three groups have no significant difference in the rates of comorbid seizure, asthma, intellectual disability, autism, and BZD/hypnotic use. The group with methylphenidate treatment for more than 180 days had the highest rate of comorbid conduct disorder (3.8%). The incidence of fracture was significantly different among three groups: 9.5% (0 day), 11.3% (1–180 days), and 8.7% (more than 180 days) respectively.

### Association between methylphenidate treatment and the risk for fracture

Analysis of methylphenidate treatment and the risk of fracture is shown in Table 2. Compared to the group without methylphenidate treatment, the group with exposure of 1–180 days did not have a reduced risk for fracture. For those received treatment lasting more than 180 days, the risk for fracture was significantly lower than the reference group (HR 0.77, CI 0.63–0.94) in multivariate analysis. Several significant risks of fracture also existed in multivariate analysis including male sex, older age, and asthma.

**Table 2:** Cox’s proportional hazards model for the risk of fracture*.

Variables	Frequency		Univariate			Multivariate		
	count	(%)	HR	95%CI	P value	HR	95%CI	P value
**Methylphenidate**								
0 day(ref.)	2,623	(42.3%)	1.00			1.00		
1–180 days	1,742	(28.1%)	1.29	1.07–1.56	0.007	1.18	0.98–1.43	0.087
>180 days	1,836	(29.6%)	0.84	0.69–1.03	0.096	0.77	0.63–0.94	0.011
**Sex**								
Female(ref.)	1,303	(21.0%)	1.00			1.00		
Male	4,898	(79.0%)	1.83	1.44–2.32	< .001	1.83	1.44–2.32	< .0001
**Age**	7.42 ± 2.65							
0–5(ref.)	1,406	(22.7%)	1.00			1.00		
6–11	4,282	(69.1%)	1.28	1.06–1.55	0.010	1.22	1.00–1.48	0.046
12–18	513	(8.3%)	2.05	1.42–2.96	< .001	1.96	1.35–2.86	0.000
**Urbanized level of residence**								
1(City) (ref.)	2,240	(36.1%)	1.00			1.00		
2	2,933	(47.3%)	1.18	0.92–1.52	0.190	1.18	0.99–1.42	0.065
3	710	(11.4%)	1.27	0.88–1.82	0.200	1.17	0.90–1.54	0.244
4(Villages)	318	(5.1%)	0.80	0.43–1.50	0.490	1.31	0.91–1.89	0.150
**Covariates**								
Seizure								
No(ref.)	5,983	(96.5%)	1.00			1.00		
Yes	218	(3.5%)	1.12	0.76–1.66	0.570	1.16	0.78–1.74	0.464
Asthma								
No(ref.)	4,207	(67.8%)	1.00			1.00		
Yes	1,994	(32.2%)	1.25	1.06–1.48	0.009	1.21	1.03–1.44	0.025
Intellectual disability								
No(ref.)	5,860	(94.5%)	1.00			1.00		
Yes	341	(5.5%)	0.78	0.53–1.15	0.212	0.81	0.55–1.21	0.306
Autism								
No(ref.)	5,935	(95.7%)	1.00			1.00		
Yes	266	(4.3%)	0.69	0.45–1.06	0.090	0.72	0.46–1.11	0.138
Conduct disorder								
No(ref.)	6,019	(97.1%)	1.00			1.00		
Yes	182	(2.9%)	0.68	0.52–1.45	0.178	0.65	0.37–1.15	0.140
BZD/hypnotic use								
No(ref.)	4,321	(69.7%)	1.00			1.00		
Yes	1,880	(30.3%)	1.13	0.95–1.33	0.170	1.05	0.88–1.24	0.624

* adjusting for sex, age, urbanized level of residence, seizure, asthma, intellectual disability, autism, conduct disorder and BZD/hypnotic use

## Discussion

To our knowledge, this is the first study that has investigated the effect of medical treatment for ADHD, and the moderational role of treatment duration, on risk for fracture. The findings revealed that methylphenidate treatment exceeding 180 days or greater was linked to nearly 25% lower risk for fracture comparing to those never received methylphenidate. In addition, shorter duration of medical treatment did not mitigate risk for fracture.

Relatively few studies have evaluated the effect of psychostimulants on bone health. The cohort study by Chou et al. reported that ADHD was associated a 1.32 times greater likelihood of fractures [22]. They also reported that compared to children without ADHD, the untreated ADHD group had a significantly increased risk for fracture (HR 1.64, 95% CI 1.37–1.96). However, amongst individuals with ADHD who were treated with stimulants, there were no significant between-group differences in risk for fracture when compared to the healthy group (HR 1.12, 95% CI 0.97–1.29). The foregoing collection of observations suggests that psychostimulant prescription mitigates the risk of fracture, but, unfortunately, data were not provided on whether the treatment duration further moderated risk reduction.

Another recent retrospective cohort study also reported that medication for ADHD was associated with lower risk of fracture [49]. They compared cases with two or more prescriptions for an ADHD medication with those without medication. Individuals without medication had a significantly increased hazard of fracture (HR: 3.9, CI: 2.6–5.9). A risk-reduction role of ADHD medication was also revealed in this study, but the role of treatment duration was still unclear.

In our study, we explored the role of the methylphenidate treatment duration on the risk for fracture. The group receiving methylphenidate for more than 180 days had significantly lower risk for fracture when compared to the group without methylphenidate treatment (HR 0.77, CI 0.63–0.94). A protective role of pharmacotherapy in the prevention of fracture was inferred. Moreover, the protective effect was observed only in cohort with exposure >180 days. Amongst individuals receiving methylphenidate for 1–180 days, the risk for fracture had no significant difference compared to those without methylphenidate treatment. That is, the treatment duration moderated the risk reduction.

Our results replicate the risk mitigation effect reported by Chou or Perry et al. Our results, however, further extend knowledge by identifying duration of exposure as a critical moderational factor. The exact mechanism was unknown. We conjecture that the salutary effects of stimulants are possibly mediated by improved cognitive function (e.g. reduced impulsivity). In previous studies, the increased risk for fracture in ADHD patients had been accounted by the core symptoms including impulsivity, recklessness, hyperactivity, and distractibility in daily life [50]. The children with ADHD have more risky behavior and might neglect the safety precaution during activities. Several interventional studies in ADHD have reported an accrual of benefit across core target symptoms in ADHD throughout sufficient duration of treatment [51]. It is possible that the medical treatment reduces the symptoms severity and further lessens the risk for fracture once the treatment duration lasts more than 180 days.

The main outcome in our study was fracture. Comparing with several recent studies on the association between ADHD, medication, and different types of injuries[14–16], our result also revealed that the ADHD medication (e.g. methylphenidate) had a possible role in injury prevention.

Other significant risks for fracture in our analysis were male sex, age, and asthma.

Male sex was associated with higher risk for fracture in our multivariate analysis (HR: 1.83, CI: 1.44–2.32). In previous literature, boys were more likely to have all injuries than girls generally [52]. Male sex had also been identified as a risk for injuries among ADHD population [53]. We had similar result regarding the sex difference in the risk for fracture.

The risk for fracture increased with ages among our study population. This finding was consistent with previous studies [53]. Among ADHD population, adolescents had a higher risk for injury than children.

In multivariate analysis, asthma was associated with higher risk for fracture (HR 1.21, CI 1.03–1.44). It was consistent with previous studies regarding the risk of fracture in asthma cases [42, 54, 55]. The exact mechanism was unclear despite several hypotheses had been proposed [56]. Corticosteroids, as one of the treatments for asthma, had detrimental effects on bone health[57]. Glucocorticoid-induced osteoporosis might be one of the possible mechanisms but further studies will be needed to clarify the exact relationship between asthma and fracture [55, 58].

The findings from our study have several important public health and therapeutic implications. From the aspect of public health, pharmacotherapy should be considered as an important strategy for preventing fracture-related dysfunction among young ADHD patient. In our study cohort, only 29.6% of ADHD cases received methylphenidate for more than 180 days. For clinicians, the need for sufficient treatment duration of pharmacotherapy should be addressed to the patients as well as their parents. Moreover, the interplay of ADHD, fracture, and pharmacotherapy merits further researches.

### Strengths and limitations

To our knowledge, this is the first study using a nationally representative sample and longitudinal dataset to investigate the relationship between methylphenidate prescription and risk for fracture. Our use of a cross-national, highly inclusive, representative database would be less susceptible to selection and recall bias. The observation that duration of exposure is significantly influenced by risk mitigation is directly consistent with the hypothesis that methylphenidate treatment is the critical intervention influencing our dependent variable.

There are several methodological limitations that affect inferences and interpretations of our data. First, the exact adherence of methylphenidate was unknown. Second, the severity of ADHD was not evaluated, quantified, and/or measured. The severity of symptoms between groups with different treatment duration could not be compared or adjusted in the following analysis. Furthermore, we are unable to ascertain whether fracture risk reduction correlates with a reduction in overall ADHD psychopathology severity. Last, several confounding factors were not available in NHIRD dataset including, but not limited to, lifestyle and body mass index. It is also unknown whether other medications or interventions had a similar impact, or moderational effects, on the fracture risk reduction seen with methylphenidate.

## Conclusion

Methylphenidate treatment decreased the risk for fracture amongst individuals with ADHD. The risk reduction is observed in individuals with longer treatment duration (i.e. >180 days). The high lifetime prevalence and persistence of ADHD, as well as the personal and economic costs associated with fracture and other unintentional injuries, underscores the public health significance of this topic. Moreover, adjudicating risk and benefits of psychostimulants in ADHD need to take into consideration both conventional measures (e.g. ADHD psychopathology), as well as the impact on real-world outcomes.
