# Correction: The association between methylphenidate treatment and the risk for fracture among young ADHD patients: A nationwide population-based study in Taiwan

**DOI:** 10.1371/journal.pone.0175617

**Published:** 2017-04-06

**Authors:** Vincent Chin-Hung Chen, Yao-Hsu Yang, Yin-To Liao, Ting-Yu Kuo, Hsin-Yi Liang, Kuo-You Huang, Yin-Cheng Huang, Yena Lee, Roger S. McIntyre, Tzu-Chin Lin

The affiliation for the tenth author is incorrect. Tzu-Chin Lin is incorrectly listed as being affiliated with **6** School of Traditional Chinese Medicine, College of Medicine, Chang Gung University, Taoyuan, Taiwan. Tzu-Chin Lin is affiliated with **7** Department of Psychiatry, Chung Shan Medical University Hospital, Taichung, Taiwan, and **8** Department of Psychiatry, School of Medicine, Chung Shan Medical University, Taichung, Taiwan.
